# Molecular Genetics Analysis of 70 Chinese Families With Muscular Dystrophy Using Multiplex Ligation-Dependent Probe Amplification and Next-Generation Sequencing

**DOI:** 10.3389/fphar.2019.00814

**Published:** 2019-07-25

**Authors:** Dong Wang, Min Gao, Kaihui Zhang, Ruifeng Jin, Yuqiang Lv, Yong Liu, Jian Ma, Ya Wan, Zhongtao Gai, Yi Liu

**Affiliations:** ^1^Pediatric Research Institute, Qilu Children’s Hospital, Shandong University, Ji’nan, China; ^2^Neurology Department, Qilu Children’s Hospital, Shandong University, Ji’nan, China

**Keywords:** muscular dystrophy, multiplex ligation-dependent probe amplification, next-generation sequencing, dystrophin (DMD), merosin-deficient congenital muscular dystrophy type 1A, LAMA2

## Abstract

**Background:** Muscular dystrophy (MD) includes multiple types, of which dystrophinopathies caused by *dystrophin* (*DMD*) mutations are the most common types in children. An accurate identification of the causative mutation at the genomic level is critical for genetic counseling of the family, and analysis of genotype–phenotype correlations, as well as a reference for the development of gene therapy.

**Methods:** Totally, 70 Chinese families with suspected MD probands were enrolled in the study. The multiplex ligation-dependent probe amplification (MLPA) was first performed to screen large deletions/duplications of *DMD* exons in the patients, and then, next-generation sequencing (NGS) was carried out to detect small mutations in the MLPA-negative patients.

**Results:** Totally, 62 mutations of *DMD* were found in 62 probands with DMD/BMD, and two compound heterozygous mutations in *LAMA2* were identified in two probands with MDC1A (a type of congenital MD), indicating that the diagnostic yield was 91.4% by MLPA plus NGS for MD diagnosis in this cohort. Out of the mutations, 51 large mutations encompassing 47 (75.8%) deletions and four duplications (6.5%) were identified by MLPA; 11 small mutations including six (9.7%) nonsense, two (3.2%) small deletions, two splice-site mutations (3.2%), and one small insertion (1.6%) were found by NGS. Large mutations were found most frequently in the hotspot region between exons 45 and 55 (70.6%). Out of the 11 patients harboring point mutations in *DMD*, 8 were novel mutations. Additionally, one novel mutation in *LAMA2* was identified. All the novel mutations were analyzed and predicted as pathogenic according to American College of Medical Genetics and Genomics (ACMG) guideline. Finally, 34 DMD, 4 BMD, 24 BMD/DMD, and 2 MDC1A were diagnosed in the cohort.

**Conclusion:** Our data indicated that the MLPA plus NGS can be a comprehensive and effective tool for precision diagnosis and potential treatment of MD and is particularly necessary for the patients at very young age with only two clinical indicators (persistent hyperCKemia and typical myopathy performance on electromyogram) but no definite clinical manifestations.

## Introduction

Muscular dystrophies (MD), an inherited group of degenerative skeletal muscle disorders, are characterized by progressive/congenital weakness and breakdown of skeletal muscles encompassing great clinical and genetic heterogeneity, and even death because of cardiomyopathy and respiratory failure (Mercuri and Muntoni, 2013; Falsaperla et al., 2016; Carter et al., 2018). Currently, MD are clinically classified to six categories with various degree of severity, including Duchenne MD (DMD) and Becker MD (BMD), limb-girdle MD (LGMD), distal MD, congenital MD (CMD), facio-scapulo-humeral MD, and myotonic MD (Falsaperla et al., 2016). DMD/BMD that are caused by mutations in X chromosome-linked *dystrophin* (*DMD*) are the most common forms in childhood with an estimated incidence of 8.3 or 7.3 per 100,000 male (Wein et al., 2015; Carter et al., 2018). DMD, a severe phenotype clinically, is characterized by a progressive loss of muscle function with onset at age 2 to 5 years, lost ambulation before age 13 years and death at approximately 20 years of age, while BMD shows a mild form with patients being loss of ambulantion after 16 years of age (Mercuri and Muntoni, 2013; Wein et al., 2015; Yiu and Kornberg, 2015). Other forms of MD are mostly autosomal recessive with rare prevalence in childhood but vary in region (Mercuri and Muntoni, 2013; Wein et al., 2015; Zimowski et al., 2017; Luce et al., 2018; Wang et al., 2019).

*DMD* is the largest gene in human, spanning 2.4 Mb of genomic region on Xp21, and containing 79 exons and 78 lengthy introns, which produces a 14.6 kb mRNA transcript (Ahn and Kunkel, 1993). To date, many mutations in *DMD* have been described, approximately 70% of which are large deletions/duplications (≥1 exon), while the remaining 25% to 30% are caused by small mutations (<1 exon), encompassing point mutations or small deletions/insertions (Yiu and Kornberg, 2015). The differences of clinical manifestations between BMD and DMD result from different mutation types in *DMD* gene. Patients will suffer from DMD when the mutation leads to a frameshift (out of frame) or generates a premature stop codon; non-functional dystrophin protein is produced. On the contrary, patients will present the phenotype of BMD when the mutation maintains in reading frame (in-frame mutation); a partially functional dystrophin protein is produced (Mohammed et al., 2018).

Recently, several promising mutation-specific molecular therapies have been developed, including exon skipping to restore the reading frame and increase expression of the compensatory dystrophin, and read-through therapy of a nonsense codon to produce full-length dystrophin which is applicable to harbor nonsense mutations for patients with DMD. Therefore, it is essential to make an early and accurate diagnosis for the patients with suspected MD to provide information on eligibility of mutation-specific treatments as well as optimal care and family planning. Multiplex ligation-dependent probe amplification (MLPA), a simple and rapid screening tool, has been developed and used to test large deletions and duplications of all 79 exons in *DMD* gene in different populations (Gatta et al., 2005; Hegde et al., 2008; Suh et al., 2017). As the small mutations are easily missed by MLPA (Stuppia et al., 2012), next-generation sequencing (NGS) has been applied for MLPA-negative patients and indicated high efficiency and cost-effectiveness but varied prevalence and mutation types in different populations of locations (Niba et al., 2014; Wang et al., 2014; Singh et al., 2018).

In this study, 70 patients clinically suspected MD and their families from Shandong province of China were investigated; MLPA was firstly used to detect large deletions and duplications in *DMD* gene in probands; then, NGS was applied to find small mutations in the MLPA-negative patients. The mutation patterns and hot spot locations were analyzed in order to establish genotype-phenotype correlations, and a reference for the development of gene therapy.

## Materials and Methods

### Patients and Samples

A total of 70 unrelated hospitalized children (67 boys and three girls, mean age 3.47 ± 2.97 years; see **Table 1** for details) with a clinically suspected diagnosis of MD and their healthy parents were enrolled in this study in Qilu Children’s Hospital of Shandong University (QCHSU) from July 2015 to December 2017, including 36 clinically suspected DMD, six suspected BMD, and 28 UMD (uncertain MD) without typical clinical phenotype due to relatively young age.

**Table 1:** The clinical and laboratory features of the 70 clinically suspected muscular dystrophy (MD) probands.

Patient ID	Sex	Age (years)	Clinical manifestation	CK value (U/L)	EMG: myopathic abnormalities	Clinical diagnosis	Genotypes
							Gene: mutation
P2	Male	7.9	Difficulty walking, Gowers sign, proximal muscle weakness, calf muscle pseudohypertrophy	16,470	NA	DMD	*DMD*: DelEX2
P3	Male	7.3	Difficulty walking, Gowers sign, difficulty climbing stairs, proximal muscle weakness	49,328	NA	DMD	*DMD*: DelEX3-13
P4	Male	1.8	Gowers sign	26,963	NA	DMD	*DMD*: DelEX3-29
P5	Female	2.4	Progressive muscle weakness	5,521	+	DMD-like	*DMD*: DelEX8-12
P6	Male	8.0	Difficulty walking, muscular hypotonia, waddling gait, falls, difficulty climbing stairs, Gowers sign	13,684	NA	DMD	*DMD*: DelEX8-23
P7	Male	2.7	Muscular hypotonia, calf muscle pseudohypertrophy	46,191	+	DMD	*DMD*: DelEX10-44
P8	Male	2.9	Progressive muscle weakness	6,093	+	DMD	*DMD*: DelEX13
P13	Male	7.3	Difficulty walking, waddling gait, falls, difficulty climbing stairs, Gowers sign	22,001	+	DMD	*DMD*: DelEX45
P18	Male	7.6	Progressive muscle weakness, falls, waddling gait	16,427	NA	DMD	*DMD*: DelEX45-54
P19	Male	1.8	Progressive muscle weakness	16,530	+	DMD	*DMD*: DelEX45-54
P23	Male	6.2	Difficulty walking, difficulty climbing stairs, calf muscle pseudohypertrophy, progressive muscle weakness	12,270	+	DMD	*DMD*: DelEX46-55
P25	Male	7.0	Waddling gait, difficulty climbing stairs, Gowers sign	12,537	NA	DMD	*DMD*: DelEX48-50
P27	Male	9.0	Difficulty walking, difficulty climbing stairs, Gowers sign, falls, waddling gait	17,713	NA	DMD	*DMD*: DelEX49-50
P30	Female	8.0	Progressive muscle weakness, calf muscle hypertrophy	13,323	NA	DMD	*DMD*: DelEX50
P31	Male	1.1	Difficulty walking	10,491	NA	DMD	*DMD*: DelEX50-52
P32	Male	3.0	Progressive muscle weakness, Gowers sign	25,758	NA	DMD	*DMD*: DelEX50-52
P35	Male	9.0	Progressive muscle weakness, difficulty climbing stairs, Gowers sign	17,771	+	DMD	*DMD*: DelEX51
P36	Male	7.0	Calf muscle pseudohypertrophy, Gowers sign	14,659	NA	DMD	*DMD*: DelEX51
P37	Male	3.2	Calf muscle pseudohypertrophy	25,596	NA	DMD	*DMD*: DelEX51-55
P38	Male	7.5	Difficulty walking, difficulty climbing stairs, Gowers sign, progressive muscle weakness, Calf muscle pseudohypertrophy	20,051	NA	DMD	*DMD*: DelEX51-55
P41	Male	4.0	Progressive muscle weakness	9,765	+	DMD	*DMD*: DelEX53-55
P43	Male	1.8	Gowers sign	10,001	+	DMD	*DMD*: DelEX55-77
P44	Male	2.0	Delayed gross motor development	7,307	+	DMD	*DMD*: DelEX56-79
P46	Male	6.7	Progressive muscle weakness, waddling gait, Calf muscle pseudohypertrophy, Gowers sign	10,578	+	DMD	*DMD*: DelEX62
P48	Male	7.4	Muscle weakness, motor delay	16,320	NA	DMD	*DMD*: DupEX3-7
P49	Male	5.6	Motor delay, muscle weakness	19,726	+	DMD	*DMD*: DupEX5-7
P50	Male	2.7	Progressive muscle weakness, difficulty climbing stairs, Gowers sign, calf muscle pseudohypertrophy	31,680	NA	DMD	*DMD*: DupEX19-21
P51	Male	7.0	Difficulty walking, Gowers sign, calf muscle pseudohypertrophy	5,243	+	DMD	*DMD*: DupEX45-54
P52	Male	2.1	Difficulty walking, calf muscle pseudohypertrophy	23,479	+	DMD	*DMD*: c.2436C > T
P53	Male	6.4	Calf muscle hypertrophy, progressive muscle weakness	15,401	NA	DMD	*DMD*: c.7264dupG
P54	Male	4.0	Progressive muscle weakness, calf muscle pseudohypertrophy	2,6917	+	DMD	*DMD*: c.1231A > T
P57	Male	3.1	Calf muscle pseudohypertrophy	8,372	+	DMD	*DMD*: c.5167G > T
P58	Male	2.3	Delayed gross motor development	35,951	+	DMD	*DMD*: 10187delC
P61	Male	7.0	Difficulty walking, Gowers sign, calf muscle pseudohypertrophy	15,795	NA	DMD	*DMD*: c.2571delC
P68	Male	5.4	Gowers sign	20,611	NA	DMD	Unknown
P69	Male	1.7	Calf muscle pseudohypertrophy	4,938	NA	DMD	Unknown
P14	Male	3.7	Calf muscle pseudohypertrophy	3,087	–	BMD	*DMD*: DelEX45-47
P16	Male	9.9	Difficulty climbing stairs, calf muscle pseudohypertrophy	5,641	NA	BMD	*DMD*: DelEX45-47
P17	Male	5.3	Calf muscle pseudohypertrophy	12,744	+	BMD	*DMD*: DelEX45-48
P56	Male	3.8	Calf muscle pseudohypertrophy	5,149	NA	BMD	*DMD*: c.1812 + 1C > T
P65	Male	5.6	Calf muscle pseudohypertrophy	13,783	+	BMD	Unknown
P70	Male	11.0	No	2,095	NA	BMD	Unknown
P1	Male	0.2	No	14,982	+	UMD	*DMD*: DelEX1-79
P9	Male	1.7	No	4,315	NA	UMD	*DMD*: DelEX26-34
P10	Male	0.4	No	20,053	NA	UMD	*DMD*: DelEX44
P11	Male	0.8	No	5,325	NA	UMD	*DMD*: DelEX44-55
P12`	Male	0.6	No	35,340	NA	UMD	*DMD*: DelEX45
P15	Male	1.0	No	4,007	NA	UMD	*DMD*: DelEX45-47
P20	Male	0.6	No	16,936	+	UMD	*DMD*: DelEX45-54
P21	Male	0.1	No	53,053	+	UMD	*DMD*: DelEX46-48
P22	Male	0.9	Calf muscle pseudohypertrophy	7,692	+	UMD	*DMD*: DelEX46-52
P24	Male	0.1	No	10,935	+	UMD	*DMD*: DelEX46-55
P26	Male	1.7	No	16,753	+	UMD	*DMD*: DelEX49-50
P28	Male	0.7	No	5,927	+	UMD	*DMD*: DelEX49-52
P29	Male	0.1	No	6,360	NA	UMD	*DMD*: DelEX49-52
P33	Male	1.2	No	16,428	+	UMD	*DMD*: DelEX51
P34	Male	0.7	No	17,001	+	UMD	*DMD*: DelEX51
P39	Male	1.0	No	8,178	+	UMD	*DMD*: DelEX52
P40	Male	0.6	No	9,606	+	UMD	*DMD*: DelEX53
P42	Male	0.8	No	4,561	+	UMD	*DMD*: DelEX55
P45	Male	0.7	No	10,444	+	UMD	*DMD*: DelEX61
P47	Male	0.1	No	21,064	+	UMD	*DMD*: DelEX69-71
P55	Male	0.9	Calf muscle pseudohypertrophy	3,676	+	UMD	*DMD*: c.580C > T
P59	Male	1.2	No	10,456	NA	UMD	*DMD*: c.7660 + 1C > G
P60	Male	1.0	No	20,926	NA	UMD	*DMD*: c.7792C > T
P62	Male	1.0	Calf muscle pseudohypertrophy	21,234	NA	UMD	*DMD*: c.6283C > T
P63	Female	0.6	Muscle weakness, muscular hypotonia, motor delay, dysphagia, weak cry	2,594	+	UMD	*LAMA2*: c.2049_2050delAG
							*LAMA2*:DelEX 4
P64	Male	0.4	Muscle weakness, muscular hypotonia, motor delay, dysphagia, weak cry	2,391	+	UMD	*LAMA2*:c.1672C > T/
							*LAMA2*:DelEX 4
P66	Male	1.7	No	2,913	NA	UMD	Unknown
P67	Male	3.0	No	4,093	NA	UMD	Unknown

All the variants in DMD gene and LAMA2 gene shown in **Table 1** **and** **Supplementary Table S1** are described using the NM_004006.2 transcript and the NM_000426.3 transcript reference sequence, respectively.

Del, deletion; Dup, duplication; EX, Exon; DMD, Duchenne’s Muscular Dystrophy; BMD, Becker Muscular Dystrophy; UMD, unclear phenotype because of young age.

NA, not available; “+”: Present; “-”: Absent.

All participants were from Han Chinese population in Shandong Province, north of China. All probands were examined and diagnosed by experienced neuromuscular specialists at Neurology Department of QCHSU. Clinical diagnosis was based on clinical features including: 1) significantly increased serum creatine kinase (CK) level; 2) myopathic abnormalities, but normal peripheral nerve conduction velocity on EMG; 3) a positive family history with MD; 4) muscular weakness; 5) difficulty in walking; 6) Gowers sign; 7) calf muscle pseudohypertrophy; 8) difficulty climbing stairs; and 9) waddling gait and so on (Mercuri and Muntoni, 2013; Carter et al., 2018).

The inclusion criteria for the probands enrolling in this study were in accord with clinical features of (1) or (1) and (2) or (1) and (2) plus one or several other features described above.

We excluded patients had no clinical feature (1). The clinical diagnosis of DMD is based on: 1) progressive symmetric muscle weakness (proximal > distal) often with calf hypertrophy, 2) symptoms present before age 5 years, and 3) lost ambulation before age 13 years. The clinical diagnosis of BMD is based on: 1) progressive symmetric muscle weakness (proximal > distal) often with calf hypertrophy, 2) activity-induced cramping (present in some individuals), 3) flexion contractures of the elbows (if present, late in the course), 4) lost ambulation after age 16 years, and 5) preservation of neck flexor muscle strength (differentiates BMD from DMD) (Darras et al., 1993). Some patients, who could not be classified in this way, were recognized as DMD when an onset of weakness occurred by the age of 5, or considered as BMD when they had very mild or nearly normal motor dysfunction after 5 years old (Marden et al., 2005).

In addition, we used “UMD” to describe the uncertain MD types of the patients who cannot be diagnosed due to very young age, only manifesting persistent hyperCKemia and typical myopathy manifestation on EMG but normal peripheral nerve conduction velocity.

**Figure 1:**
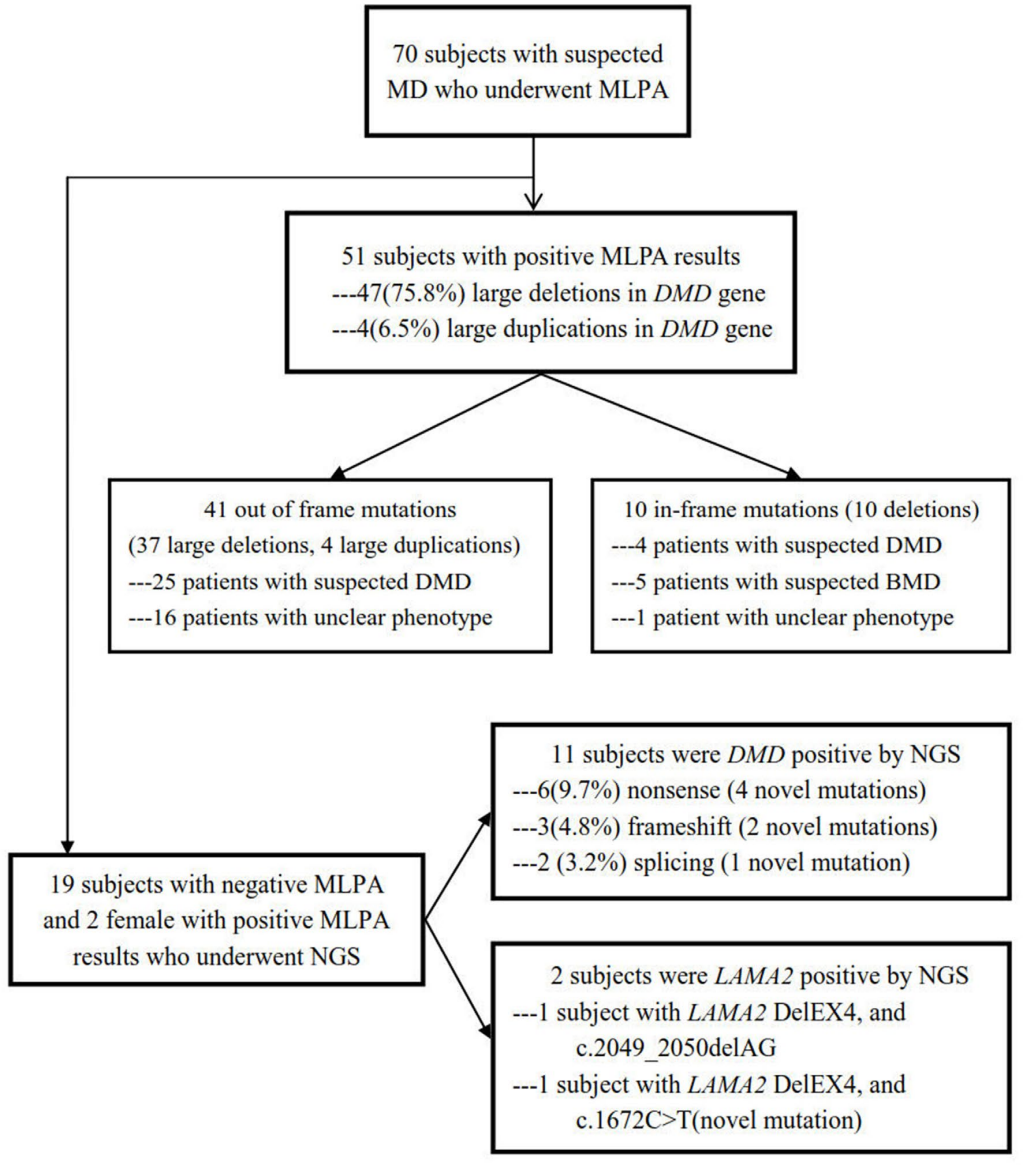
Flow chart of this study design. A total of 70 subjects with suspected MD underwent MLPA, and 51 were positive including 41 out of frame mutations and 10 in-frame mutations, while in the rest of the 19 subjects with negative MLPA and two female with positive MLPA, NGS was performed, with 11 positive in *DMD* and 2 positive in *LAMA2*.

Blood samples obtained from the subjects were collected in EDTA vacutainer, and genomic DNA was extracted by using QIAamp DNA Blood Mini Kit (Qiagen, Shanghai, China) following the manufacturer’s instructions. MLPA was used as the first-line molecular detection, and NGS was applied to detect small mutations in MLPA-negative samples (The work flow chart of the study was shown in **Figure 1**).

### MLPA Assay

The MLPA reaction was carried out to screen all exons of *DMD* using SALSA MLPA probe sets P034 and P035 (MRC-Holland, Amsterdam, the Netherlands) according to the manufacturer’s instructions. Amplified products were separated using a 3500 XL Genetic Analyzer (ABI, Carlsbad, CA, USA), and the data were analyzed by Coffalyser Software (MRC-Holland, Amsterdam, the Netherlands).

### Next-Generation Sequencing

Totally, 1,500 ng genomic DNA was fragmented to an average size of 300 bp; then, the fragmented genomic DNA was used to prepare sequencing libraries and 8 bp barcoded sequencing adapters were ligated to the DNA fragments before final hybridization with SureSelect captured Exome probes (Agilent, Santa Clara, CA, USA). The quality and quantity of the libraries were assessed by both Advanced Analytical Technologies Inc. (AATI) Fragment Analyzer (Ankeny, Des Moines, IA, USA) and qPCR. Purified sequencing libraries were pooled together and massively parallel-sequenced using Illumina HiSeq X Ten platform yielding an average of 1.5 Gb of total sequence per sample at an average sequencing depth of 100×. The data were then aligned to version GRCh37/hg19 of the human genome in NextGENe Software v2.3.4 (SoftGenetics, State College, PA, USA) that aligned sequence reads to the reference. NextGENe Software uses a preloaded index alignment algorithm that employs a suffix array that is represented by the Burrows-Wheeler Transform (BWT).

Both the general pipelines for NGS data analyses of diseases and data analysis strategies for the pathogenic genes of MD were conducted according to the recommendations from Jin et al. (2018). Variants were classified according to the 2015 ACMG guideline for the interpretation of sequence variants. All the mutations that can potentially cause the diseases were verified by Sanger sequencing on a 3130 Genetic Analyzer (ABI, Carlsbad, CA, USA).

### Validation of Gene Mutations

Quantitative PCR (qPCR) and Sanger sequencing were then utilized to validate the potentially pathogenic variants of exon deletions/duplications and small mutations in the patients with designed specific primers. The qPCR was amplified according to the manufacturer’s recommendations on a real-time PCR system (LightCycler 480 II, Roche, Foster City, USA). Copy number variations were determined based on the ratio of copies of the deletion fragment to a reference gene (*GAPDH*) with the SYBR Premix Ex Taq II PCR reagent kit (TakaRa Bio, Dalian, China) according to the manufacturer’s protocol. The PCR products were puriﬁed and sequenced using an ABI Prism 3700 automated sequencer (Applied Biosystems, Foster City, CA).

### Statistical Analysis

SPSS 17.0 (IBM, Armonk, NY, United States) was used for statistical analyses. Two-sided Fisher’s test was used to compare CK value between BMD and UMD. Correlations between phenotypes and factors (age of examination, mutation type, and CK values as mean ± SD) were analyzed using logistic regression. It was considered statistically significant when *P* value was less than 0.05, and the confidence interval was 95%.

## Results

### Clinical Findings

In total, 70 Chinese families with 70 clinically suspected MD patients were enrolled in the study, including 42 with a clinically suspected DMD/BMD (36 suspected DMD and 6 suspected BMD), and 28 (named as UMD, mean age 0.64 ± 0.34 years) lack of clinical phenotype due to relatively young age, with only persistent hyperCKemia and typical myopathy manifestation on EMG but normal peripheral nerve conduction velocity.

The average CK values for these patients clinically diagnosed with DMD and BMD were 17,528.33 ± 10,234.82 U/L and 6,017.71 ± 2,890.50 U/L, respectively, which indicated a significant difference between both categories (*P* < 0.01) (**Table 1**).

### Detection of Mutations in *DMD* by MLPA

**Figure 2:**
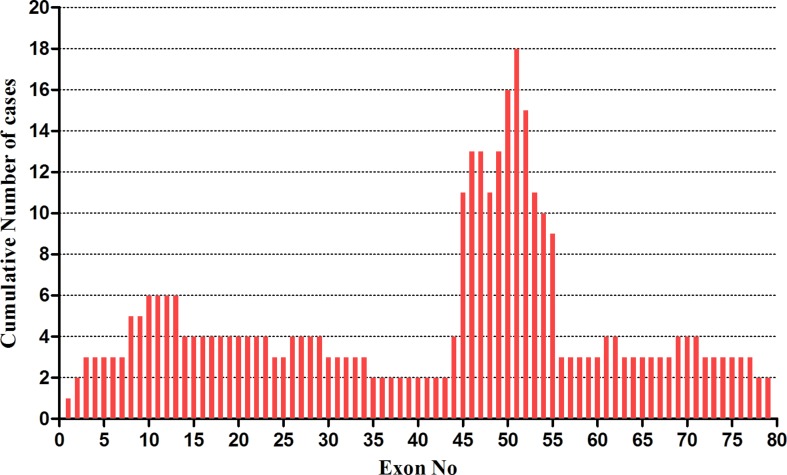
The distribution of gross deletions identified in *DMD* gene by MLPA. The frequency of large deletions for each exon of *DMD* gene was shown. The abscissa showed exon number of *DMD* gene from exons 1 to 79; the ordinate demonstrated the frequency of large deletions for each exon. A region with a highest frequency of deletions was found in exons 45–55.

Large rearrangements in *DMD* were detected in the 70 probands using MLPA and validated by qPCR. A total of 51 (72.9%) deletions and duplications were found in 51 patients including two girls, 47 (75.8%) of which were deletions, and 4 were duplications (6.5%). Overall, 38 different rearrangements were identified. Among the 51 positive results, 41 showed out of frame mutations (37 large deletions, 4 large duplications) and 10 showed in-frame mutations (deletions). Of the 41 out of frame mutations, 25 were found in clinically diagnosed DMD patients, and 16 in very young UMD patients. Among the patients with 10 in-frame mutations, 5 BMD, 4 DMD, and 1 UMD were finally determined after reviewing their clinical manifestations (**Figure 1**, **Table 1**). There were 11 different single-exon deletions in *DMD* gene identified in 15 patients involving exons 2, 13, 44, 45, 50, 51, 52, 53, 55, 61, and 62, respectively, while 36 cases were found to have multiple exon deletions. The largest deletion covering the whole exons from 1 to 79 in *DMD* was found in a 2-month-old male DMD patient (P1) with a higher CK level of 14,982 U/L (**Table 1**). The hotspot region was demonstrated between exons 45 and 55 in which 70.6% large deletions were found most frequently, followed by deletions in exons 3–34 (24.4%) (**Figure 2**); three out of four duplications in *DMD* were detected in the proximal hotspot regions between exons 3 and 25 (**Table 1**). However, 19 cases failed to detect large deletions or duplications in *DMD* by MLPA.

### Detection of Small Mutations Using NGS

NGS were then utilized to detect small mutations associated with MD in the 19 MLPA-negative patients as well as the two female patients showing MLPA-positive (P5 and P30; see **Figure 1** and **Table 1** for details). Sanger sequencing was used to validate the potentially pathogenic small mutations in the patients and carriers. Overall, 11 point mutations in *DMD* were found in 11 different probands, respectively, which were 3 *de novo* and 8 maternally inherited, including 6 (9.7%) nonsense mutations, 2 (3.2%) small deletions, 2 splice-site mutations (3.2%), and 2 small insertions (1.6%) (**Figure 1**, **Table 2**). The point mutations of c.2436C > T, c.7264dupG, c.1231A > T, c.5167G > T, 10187delC, c.7660+1C > G, and c.7792C > T in the patients of P52, P53, P54, P57, P58, P59, and P60 were novel, unreported previously. No small mutations were found in the two MLPA-positive female patients. Meanwhile, the known pathogenic mutation c.2049_2050delAG and novel mutation c.1672C > T in *LAMA2* were detected in two patients of P63 and P64, respectively (**Figure 1**, **Table 2**). All the *de novo* mutations were finally predicted to be pathogenic after analysis according to ACMG guideline (**Table 2**).

**Table 2:** Summary of putative pathogenic mutations in *DMD*/*LAMA2* gene analyzed by NGS and validated by Sanger sequencing.

Patient ID	Gene/exon	DNA change	Effect	Mutation type	dbSNP/1000G/EVS/ExAC	Status	Inheritance	Pathogenicity prediction score		Pathogenic evaluation according to ACMG
								Mutation Taster	Human Splicing Finder	
P52	*DMD/*20	c.2436C > T	p.Trp812Ter	Nonsense	0/0/0/0	Novel	De novo	1		Pathogenic
P53	*DMD/*50	c.7264dupG	p.Ala2422GlyfsTer5	Frameshift	0/0/0/0	Novel	Maternal	1		Pathogenic
P54	*DMD/*11	c.1231A > T	p.Lys411Ter	Nonsense	0/0/0/0	Novel	Maternal	1		Pathogenic
P55	*DMD/*7	c.580C > T	p.Gln194Ter	Nonsense	0/0/0/0	Flanigan et al., 2009	Maternal	1		Pathogenic
P56	*DMD/*15	c.1812+1C > T	–	Splicing	0/0/0/0	Piton et al., 2013; Taylor et al., 2007	Maternal	1	1	Pathogenic
P57	*DMD/*37	c.5167G > T	p.Glu1723Ter	Nonsense	0/0/0/0	Novel	De novo	1		Pathogenic
P58	*DMD/*70	10187delC	p.Pro3396GlnfsTer6	Frameshift	0/0/0/0	Novel	Maternal	1		Pathogenic
P59	*DMD/*52	c.7660+1C > G	–	Splicing	0/0/0/0	Novel	Maternal	1	1	Pathogenic
P60	*DMD/*53	c.7792C > T	p.Gln2598Ter	Nonsense	0/0/0/0	Novel	De novo	1		Pathogenic
P61	*DMD/*20	c.2571delC	p.Pro857ProfsTer14	Frameshift	0/0/0/0	Li et al., 2015	Maternal	1		Pathogenic
P62	*DMD/*43	c.6283C > T	p.Arg2095Ter	Nonsense	0/0/0/0	Cho et al., 2017;	Maternal	1		Pathogenic
P63	*LAMA2/*exon14	c.2049_2050delAG	p.Arg683SerfsTer21	Nonsense	0/0/0/0		Paternal	1		Pathogenic
	LAMA2/exon 4	DelEX 4	–	In-frame	0/0/0/0		Maternal			Pathogenic
P64	LAMA2/exon 4	DelEX 4	–	In-frame	0/0/0/0		Maternal			Pathogenic
	LAMA2/exon 12	c.1672C > T	p.Gln558Ter	Nonsense	0/0/0/0	Novel	Paternal	1		Pathogenic

All the variants shown in **Table 2** are described using the NM_004006.2 of DMD/NM_000426.3 of LAMA2 transcript references, respectively.

del, deletion; dup, duplication; fs, frameshift; Ter, termination; in-frame, in-frame mutation.

ACMG, American College of Medical Genetics and Genomics.

### Confirmation of *LAMA2* Mutations in Two MD Patients

To further analyze and confirm the possible deletion in *LAMA2* in both patients of P63 and P64, MLPA was performed for detection of *LAMA2* deletion using SALSA MLPA probe sets P391 (MRC-Holland, Amsterdam, the Netherlands) and confirmed that two patients of P63 and P64 harbored DelEX 4 in *LAMA2*. Thus, both compound heterozygous mutations in *LAMA2* were detected in patients of P63 and P64, individually, with P63 carrying c.2049_2050delAG and DelEX 4, whereas P64 having c.1672C > T and DelEX 4, inherited from father and mother, respectively (**Table 2**). After analyzing and comparing the results with the databases, c.1672C > T (p.Arg2095Ter) was determined as a novel unreported mutation. The clinical symptoms of the two patients showed that they got apparent muscle weakness since the first 6 months of life, hypotonia, poor suck and cry, and delayed motor development, while their parents had normal phenotype. Moreover, clinical laboratory tests showed significantly increased serum CK levels (**Table 1**) and typical pattern of myopathy on EMG. So, the two patients were finally diagnosed as Merosin-deficient congenital MD type 1A (MDC1A).

### Data Summary

**Figure 3:**
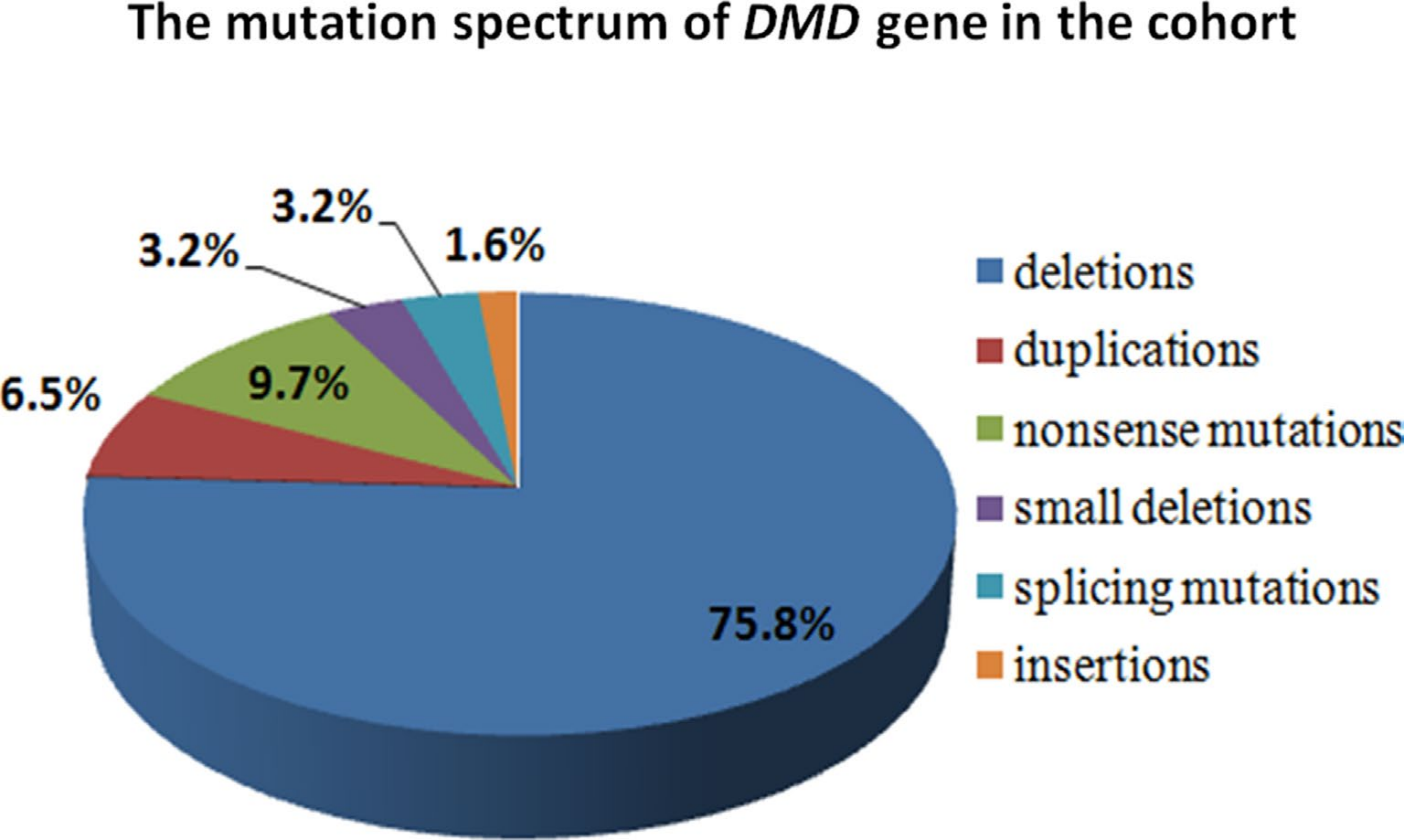
The mutation spectrum of *DMD* gene in the cohort. The mutation spectrum of *DMD* gene in the cohort was shown, including 75.8% large deletions, 6.5% large duplications, 9.7% nonsense mutations, 3.2% small deletions, 3.2% splice-site mutations, and 1.6% small insertion.

A total of 70 cases were included in this study, of which 62 were *DMD* gene mutations-positive, and 2 were *LAMA2* mutations-positive detected by MLPA plus NGS. Large deletions and duplications in *DMD* gene were detected in 51 patients by MLPA, of which deletions were found in 47 cases, and duplications were in 4 cases. The remaining 19 cases who were MLPA-negative undergone NGS, and 11 small mutations were identified in 15 male cases. All genetic mutations in *DMD* were shown in **Table 2**. The overall positive mutation rate was 91.4% (64/70), encompassing 47 (75.8%) large deletions, 4 (6.5%) large duplications, 6 (9.7%) nonsense mutations, 2 (3.2%) small deletions, 2 (3.2%) splice-site mutations, and 1 (1.6%) small insertion (**Figure 3**).

Final diagnosis was made based on the phenotypes and genotypes in the 70 patients. Of them, 34 DMD, 4 BMD, and 2 MDC1A were made. Additional 24 cases couldn’t be differentiated between BMD and DMD due to very young age at present and were diagnosed as BMD/DMD. More research in the future need to be done in the remaining six undiagnosed patients (**Table 3**).

**Table 3:** Final diagnosis of all 70 patients after detection with MLPA and NGS.

Final diagnosis		Genotype	
Disease	Case no. %	Gene: mutation	Case no. %
DMD	P34, 48.6%	*DMD*: large deletion	24, 34.3%
		*DMD*: large duplication	4, 5.7%
		*DMD*: small mutation	6, 8.6%
BMD	P4, 5.7%	*DMD*: large deletion	3, 4.3%
		*DMD*: small mutation	1, 1.4%
BMD/DMD*	P24, 34.3%	*DMD*: large deletion	20, 28.6%
		*DMD*: small mutation	4, 5.7%
MDC1A	P2, 2.9%	*LAMA2*: large deletion	2, 2.9%
		*LAMA2*: small mutation	2, 2.9%
Undiagnosed^¶^	P6, 8.6%	No mutation found	6, 8.6%

*Could not differentiate from each other at present due to very young age of the patients.

^¶^Needs to do more research.

## Discussion

MDs are a clinically, genetically, and biochemically heterogeneous group of degenerative skeletal muscle disorders. As one of children’s rare diseases, diagnosis of MDs still faces many challenges in China (Fang et al., 2017; Ni and Shi, 2017). DMD/BMD are the most common types of MD in childhood and caused by the loss of dystrophin function completely or partially (Wein et al., 2015; Yiu and Kornberg, 2015). Muscle biopsy, an invasive technique, showing a specific absence of dystrophin protein in DMD patients while partially functional dystrophin protein in BMD, became the golden standard test in diagnosing MD after the 1960s (Vogel and Zamecnik, 2005; Skram et al., 2009). With the development and availability of genetic techniques, the muscle biopsy has been gradually replaced by gene tests that become the new golden standard as stated in the latest guideline (Birnkrant et al., 2018). MLPA has been used to detect large deletion/duplication (≥1 exon) of *DMD* in roughly 70% of cases, and NGS is applied to detect the remaining 25% to 30% small mutations (<1 exon), such as point mutations and small deletions or insertions, which indicated that MLPA is a simple, rapid, and reliable technique for detection of deletion/duplication in *DMD* gene (Yiu and Kornberg, 2015; Wein et al., 2015; Suh et al., 2017). Moreover, MLPA has been suggested to use as a first-line screening test for detection of DNA rearrangements in *DMD* in clinically suspected DMD/BMD patients (Mohammed et al., 2018).

In this study, MLPA was firstly applied to detect deletions and duplications in *DMD* in 70 patients with suspected MD, and 38 DNA rearrangements in *DMD* gene were detected in 51 (72.9%) patients (comprised 31 suspected DMD/BMD and 20 UMD). Out of the mutations, 47 large deletions (47/70, 67.14%) and 4 large duplications (4/70, 5.71%) were found, indicating a higher proportion of *DMD* deletion in comparison with *DMD* duplication, which is similar as the most previous studies (Danieli et al., 1993; Muntoni et al., 2003; Chen et al., 2014; Deepha et al., 2017; Okubo et al., 2017; Suh et al., 2017) but different from some reports that demonstrated about 40% deletion and about 25% duplication (Hwa et al., 2007; Wang et al., 2008; Lee et al., 2012). So, further investigation of the differences in different populations from various locations still needs to be done.

Despite distribution of deletions and duplications occurring in almost every exon in *DMD* gene, two deletion hot spot regions with a non-random style have been repeatedly reported at the 5’-end and the central region around exons 44 to 55 (Muntoni et al., 2003; Aartsma-Rus et al., 2006; Suh et al., 2017; Mohammed et al., 2018). In our cohort, we could detect the deletion hot spot in the central region of exons 45 to 55, with a distribution rate in this region of approximately 70.6%, which is in accordance with previous studies (Danieli et al., 1993; Hwa et al., 2007; Lee et al., 2012; Chen et al., 2014; Okubo et al., 2017). Out of four duplications detected in this study, three showed in the proximal hot spot region from exons 3 to 25 similar as the previous report (Okubo et al., 2017).

With the great advantage of cost-effectiveness and accuracy, NGS has made the detection of small mutations in *DMD* revolutionized (Zhang et al., 2019; Jia and Shi, 2017). To minimize the cost of NGS for detection of mutations, we utilized NGS in the remaining 19 MLPA-negative (13 DMD/BMD and 6 UMD) patients and the two female patients of MLPA-positive. In total, 13 small mutations in 13 (7 DMD/BMD, 6 UMD) patients were found, including 11 *DMD* mutations (three *de novo* and eight maternal inheritances) and 2 *LAMA2* mutations. Among 11 point mutations of *DMD* gene, 6 were nonsense mutations (54.55%), which were the most common point mutations in this study, similar to the report (Okubo et al., 2017), and then followed by splice-site mutations (18.2%), small deletions (18.2%), and small insertions (9.1%); however, no missense mutations were detected in this cohort, which might be due to the small sample size. The distribution of mutations types (six nonsense, three frameshift, and two splicing) in this study was consistent with some studies using different methods (Flanigan et al., 2009; Takeshima et al., 2010; Lim et al., 2011). As expected, G:C to A:T transitions were the most prevalent stop mutation class; our results showed that the transversions of GC to AT accounted for 63.63% (7/11). After mutation verification of the probands’ mothers, eight novel (seven in *DMD* and one in *LAMA2*) mutations were identified. All these novel mutations were predicted to generate a premature stop codon in the coding sequence of *DMD* causing premature termination of the protein product lacking key domains of dystrophin protein, and produce a non-functional dystrophin protein thereby leading to DMD. In this way, we got much higher detection rate of 91.4% than muscle biopsies with more precise and sensitive diagnosis of MD.

Two unrelated female patients clinically diagnosed as DMD-like MD (**Table 1**), whose parents showed normal phenotypes, were detected to carry a heterozygous deletion of exon 50 and exons 8–12 in *DMD* by MLPA, inherited maternally, but no disease-causing small mutations were found by the NGS. As we know, most heterozygous *DMD* mutations’ female carriers were asymptomatic, however, although of which 2.5–7.8% were symptomatic with symptoms ranging from mild muscle weakness to a rapidly progressive DMD-like MD (Moser and Emery, 1974; Norman and Harper, 1989; Taylor et al., 2007). Fujii et al. (2009) summarized the mechanism for female DMD and BMD as the following five cases: uniparental disomy of the entire X chromosome with mutations, skewed X inactivation either balanced X-autosome translocation patients or in the female carriers with *dystrophin* mutation, Turner syndrome with a *dystrophin* mutation on the remaining X chromosome, co-occurrence of mutations in both *dystrophin* and androgen receptor genes, and double *dystrophin* mutations on both X chromosomes. In regard of the two female cases in this study, the most probable mechanism is skewed X inactivation in the female *dystrophin* mutation carriers. The further X chromosome inactivation analyses of the two patients are required to confirm the inference.

In this cohort, 28 UMD patients presented only persistent hyperCKemia (CK from 2,391 to 35,340 U/L) and typical myopathy performance on EMG but without typical clinical features due to very young age (average age at 0.64 ± 0.34 years). After detecting mutation using the MLPA plus NGS, 24 mutations in *DMD* were found, including 20 large deletions and 3 nonsense and 1 splice-site mutations. Additionally, two small mutations in *LAMA2* were detected. *Laminin-α2* (*LAMA2*) is the causative gene to MDC1A (merosin-deficient congenital MD type 1A), which is a type of CMD, an autosomal recessive disorder. So far, more than 493 mutations in *LAMA2* gene have been listed in the locus specific database (LSDB) (April, 2018), in which a limited number of large deletions/duplications have been reported. In this study, deletions of exon 4 in *LAMA2* in the two patients with MDC1A were detected, respectively, which have been reported in five other Chinese patients by Xiong et al. (2015), but have not been found in other countries. So, we inferred that it might be a particular mutation type in Chinese population. In addition, the mutation c.2049_2050delAG in *LAMA2* in patient P63 was a known pathogenic mutation (Guicheney et al., 1998), while c.1672C > T (p.Q558X) in *LAMA2* in patient P64 was a *de novo* nonsense mutation, unreported in population databases (ExAC, dbSNP, and 1,000 genomes), leading to a truncated and nonfunctional laminin-α2 protein, which was also predicted to be pathogenic by using MutationTaster. Moreover, many pathogenic truncating mutations downstream of this mutation site have been reported in the LSDB for the *LAMA2* gene (http://www.lovd.nl/LAMA2). Thus, we inferred that the mutation c.1672C > T (p.Q558X) in *LAMA2* was probably pathogenic.

To the best of our knowledge, this is the first report using the molecular genetics techniques to identify genes’ mutations in young patients with MD before their clinical features appeared. And it is critical to perform molecular genetics detection for the UMD patients with indicators like persistent hyperCKemia and typical myopathy performance on EMG but without definite clinical manifestations at a relatively young age. The genotype-first approach can provide a definitive diagnosis mainly based on molecular evidence (Shen, 2018). This study of using MLPA plus NGS provides a comprehensive and effective genetic approach for precise and early diagnosis of MD, with the diagnostic yield of 91.4% (64/70).

## Conclusions

In this study, we identified 51 large deletions/duplications, 11 small mutations in *DMD* gene, and 2 mutations in *LAMA2* gene by MLPA plus NGS. We finally diagnosed 34 DMD, 4 BMD, 24 BMD/DMD, and 2 MDC1A in the cohort. Our data indicated that the MLPA plus NGS can be a comprehensive and effective tool for precision diagnosis of MD and is particularly necessary for the patients with only two clinical indicators (persistent hyperCKemia and typical myopathy performance on electromyogram) but no definite clinical manifestations at a relatively young age, which would benefit the very young patients for early diagnosis and treatment as well. Moreover, the method is suitable for early precise diagnosis of children with unclear clinical subtypes of MD.

## Ethics Statement

This study was approved by Ethics Committee of Qilu Children’s Hospital of Shandong University. Informed written consent has been provided by the guardians of the patients. All the patients’ information was submitted anonymously. All the procedures performed in the study were in accordance with the Declaration of Helsinki.

## Author Contributions

The study was conceived and designed by ZG and YiL. The experiments were conducted by DW, MG, KZ, YuL, JM and YW. Data analyzed by DW, MG, KZ and YL. RJ, YoL and ZG contributed clinical diagnosis of the patients. The paper was written by DW and YL.

## Funding

This work was funded by Development Project of Science and Technology in Shandong Province (2013GSF11829) and Jinan outstanding scientific and technological innovation team fund (20150519). The funders had no role in study design, data collection and analysis, decision to publish, or preparation of the manuscript.

## Conflict of Interest Statement

The authors declare that the research was conducted in the absence of any commercial or financial relationships that could be construed as a potential conflict of interest.

## References

[B1] Aartsma-Rus A. Van Deutekom J. C. Fokkema I. F. Van Ommen G. J. Den Dunnen J. T. (2006). Entries in the Leiden Duchenne muscular dystrophy mutation database: an overview of mutation types and paradoxical cases that confirm the reading-frame rule. Muscle Nerve 34 (2), 135–144. 10.1002/mus.20586 16770791

[B2] Ahn A. H. Kunkel L. M. (1993). The structural and functional diversity of dystrophin. Nat. Genet. 3 (4), 283–291. 10.1038/ng0493-283 7981747

[B3] Birnkrant D. J. Bushby K. Bann C. M. Apkon S. D. Blackwell A. Brumbaugh D. (2018). Diagnosis and management of Duchenne muscular dystrophy, part 1: diagnosis, and neuromuscular, rehabilitation, endocrine, and gastrointestinal and nutritional management. Lancet Neurol. 17, 251–267. 10.1016/S1474-4422(18)30024-3 29395989 PMC5869704

[B4] Carter J. C. Sheehan D. W. Prochoroff A. Birnkrant D. J. (2018). Muscular dystrophies. Clin. Chest Med. 39 (2), 377–389. 10.1016/j.ccm.2018.01.004 29779596

[B5] Chen C. Ma H. Zhang F. Chen L. Xing X. Wang S. (2014). Screening of Duchenne muscular dystrophy (DMD) mutations and investigating its mutational mechanism in Chinese patients. PLoS One 9 (9), e108038. 10.1371/journal.pone.0108038 25244321 PMC4171529

[B6] Cho A. Seong M. W. Lim B. C. Lee H. J. Byeon J. H. Kim S. S. (2017). Consecutive analysis of mutation spectrum in the dystrophin gene of 507 Korean boys with Duchenne/Becker muscular dystrophy in a single center. Muscle Nerve 55 (5), 727–734. 10.1002/mus.25396 27593222

[B7] Danieli G. A. Mioni F. Muller C. R. Vitiello L. Mostacciuolo M. L. Grimm T. (1993). Patterns of deletions of the dystrophin gene in different European populations. Hum. Genet. 91 (4), 342–346. 10.1007/BF00217354 8099058

[B8] Darras B. T. Urion D. K. Ghosh P. S. (1993). “Dystrophinopathies,” in GeneReviews((R)). Eds. Adam M. P. Ardinger H. H. Pagon R. A. Wallace S. E. Bean L. J. H. Stephens K. Amemiya A. Seattle, WA: University of Washington, Seattle.

[B9] Deepha S. Vengalil S. Preethish-Kumar V. Polavarapu K. Nalini A. Gayathri N. (2017). MLPA identification of dystrophin mutations and in silico evaluation of the predicted protein in dystrophinopathy cases from India. BMC Med. Genet. 18 (1), 67. 10.1186/s12881-017-0431-6 28610567 PMC5470271

[B10] Fang F. Liu Z. Fang H. Wu J. Shen D. Sun S. (2017). The clinical and genetic characteristics in children with mitochondrial disease in China. Sci. China Life Sci. 60 (7), 746–757. 10.1007/s11427-017-9080-y 28639102

[B11] Falsaperla R. Pratico A. D. Ruggieri M. Parano E. Rizzo R. Corsello G. (2016). Congenital muscular dystrophy: from muscle to brain. Ital. J. Pediatr. 42 (1), 78. 10.1186/s13052-016-0289-9 27576556 PMC5006267

[B12] Flanigan K. M. Dunn D. M. von Niederhausern A. Soltanzadeh P. Gappmaier E. Howard M. T. (2009). Mutational spectrum of DMD mutations in dystrophinopathy patients: application of modern diagnostic techniques to a large cohort. Hum. Mutat. 30 (12), 1657–1666. 10.1002/humu.21114 19937601 PMC3404892

[B13] Fujii K. Minami N. Hayashi Y. Nishino I. Nonaka I. Tanabe Y. (2009). Homozygous female Becker muscular dystrophy. Am. J. Med. Genet. A 149A (5), 1052–1055. 10.1002/ajmg.a.32808 19396825

[B14] Gatta V. Scarciolla O. Gaspari A. R. Palka C. De Angelis M. V. Di Muzio A. (2005). Identification of deletions and duplications of the DMD gene in affected males and carrier females by multiple ligation probe amplification (MLPA). Hum. Genet. 117 (1), 92–98. 10.1007/s00439-005-1270-7 15841391

[B15] Guicheney P. Vignier N. Zhang X. He Y. Cruaud C. Frey V. (1998). PCR based mutation screening of the laminin alpha2 chain gene (LAMA2): application to prenatal diagnosis and search for founder effects in congenital muscular dystrophy. J. Med. Genet. 35 (3), 211–217. 10.1136/jmg.35.3.211 9541105 PMC1051244

[B16] Hegde M. R. Chin E. L. Mulle J. G. Okou D. T. Warren S. T. Zwick M. E. (2008). Microarray-based mutation detection in the dystrophin gene. Hum. Mutat. 29 (9), 1091–1099. 10.1002/humu.20831 18663755 PMC2574813

[B17] Human Gene Mutation Database [Online]. Available: https://portal.biobase-international.com/cgi-bin/portal/login.cgi [Accessed].

[B18] Hwa H. L. Chang Y. Y. Chen C. H. Kao Y. S. Jong Y. J. Chao M. C. (2007). Multiplex ligation-dependent probe amplification identification of deletions and duplications of the Duchenne muscular dystrophy gene in Taiwanese subjects. J. Formos. Med. Assoc. 106 (5), 339–346. 10.1016/S0929-6646(09)60318-1 17561468

[B19] Jia J. Shi T. (2017). Towards efficiency in rare disease research: what is distinctive and important? Sci. China Life Sci. 60 (7), 686–691. 10.1007/s11427-017-9099-3 28639105

[B20] Jin Y. Zhang L. Ning B. Hong H. Xiao W. Tong W. (2018). Application of genome analysis strategies in the clinical testing for pediatric diseases. Pediatr. Invest. 2 (2), 72–81. 10.1002/ped4.12044 PMC6089540 30112248

[B21] Lee B. L. Nam S. H. Lee J. H. Ki C. S. Lee M. Lee J. (2012). Genetic analysis of dystrophin gene for affected male and female carriers with Duchenne/Becker muscular dystrophy in Korea. J. Korean Med. Sci. 27 (3), 274–280. 10.3346/jkms.2012.27.3.274 22379338 PMC3286774

[B22] Li X. Zhao L. Zhou S. Hu C. Shi Y. Shi W. (2015). A comprehensive database of Duchenne and Becker muscular dystrophy patients (0-18 years old) in East China. Orphanet. J. Rare Dis. 10, 5. 10.1186/s13023-014-0220-7 25612904 PMC4323212

[B23] Lim B. C. Lee S. Shin J. Y. Kim J. I. Hwang H. Kim K. J. (2011). Genetic diagnosis of Duchenne and Becker muscular dystrophy using next-generation sequencing technology: comprehensive mutational search in a single platform. J. Med. Genet. 48 (11), 731–736. 10.1136/jmedgenet-2011-100133 21969337

[B24] Luce L. N. Carcione M. Mazzanti C. Ferrer M. Szijan I. Giliberto F. (2018). Small mutation screening in the DMD gene by whole exome sequencing of an argentine Duchenne/Becker muscular dystrophies cohort. Neuromuscul. Disord. 28 (12), 986–995. 10.1016/j.nmd.2018.08.012 30342905

[B25] Marden F. A. Connolly A. M. Siegel M. J. Rubin D. A. (2005). Compositional analysis of muscle in boys with Duchenne muscular dystrophy using MR imaging. Skeletal Radiol. 34 (3), 140–148. 10.1007/s00256-004-0825-3 15538561

[B26] Mercuri E. Muntoni F. (2013). Muscular dystrophies. Lancet 381, 845–860. 10.1016/S0140-6736(12)61897-2 23465426

[B27] Moser H. Emery A. E. (1974). The manifesting carrier in Duchenne muscular dystrophy. Clin. Genet. 5 (4), 271–284. 10.1111/j.1399-0004.1974.tb01694.x 4854942

[B28] Mohammed F. Elshafey A. Al-Balool H. Alaboud H. Ben Ali M. Bager A. (2018). Mutation spectrum analysis of Cuchenne/Becker muscular dystrophy in 68 families in Kuwait: the era of personalized medicine. PLoS One 13 (5), e0197205. 10.1371/journal.pone.0197205 29847600 PMC5976149

[B29] Muntoni F. Torelli S. Ferlini A. (2003). Dystrophin and mutations: one gene, several proteins, multiple phenotypes. Lancet Neurol. 2, 731–740. 10.1016/S1474-4422(03)00585-4 14636778

[B30] Ni X. Shi T. (2017). The challenge and promise of rare disease diagnosis in China. Sci. China Life Sci. 60 (7), 681–685. 10.1007/s11427-017-9100-1 28623543

[B31] Niba E. T. Tran V. K. Tuan-Pham le A. Vu D. C. Nguyen N. K. Ta V. T. (2014). Validation of ambiguous MLPA results by targeted next-generation sequencing discloses a nonsense mutation in the DMD gene. Clin. Chim. Acta 436, 155–159. 10.1016/j.cca.2014.05.018 24892813

[B32] Norman A. Harper P. (1989). A survey of manifesting carriers of Duchenne and Becker muscular dystrophy in Wales. Clin. Genet. 36 (1), 31–37. 10.1111/j.1399-0004.1989.tb03363.x 2766561

[B33] Okubo M. Goto K. Komaki H. Nakamura H. Mori-Yoshimura M. Hayashi Y. K. (2017). Comprehensive analysis for genetic diagnosis of Dystrophinopathies in Japan. Orphanet J. Rare Dis. 12 (1), 149. 10.1186/s13023-017-0703-4 28859693 PMC5580216

[B34] Piton A. Redin C. Mandel J. L. (2013). XLID-causing mutations and associated genes challenged in light of data from large-scale human exome sequencing. Am. J. Hum. Genet. 93 (2), 368–383. 10.1016/j.ajhg.2013.06.013 23871722 PMC3738825

[B35] Skram M. K. Gulati S. Larsson E. Lindal S. Torp S. H. (2009). Muscle biopsies in children—an evaluation of histopathology and clinical value during a 5-year period. Ups J. Med. Sci. 114 (1), 41–45. 10.1080/03009730802604949 19242871 PMC2852747

[B36] Shen Y. (2018). Next-generation sequencing based molecular testing is an equalizer for diagnostic service of rare genetic disorders in China. Pediatr. Invest. 2 (2), 96–97. 10.1002/ped4.12036 PMC7331406 32851240

[B37] Singh B. Mandal K. Lallar M. Narayanan D. L. Mishra S. Gambhir P. S. (2018). Next generation sequencing in diagnosis of MLPA negative cases presenting as duchenne/becker muscular dystrophies. Indian J. Pediatr. 85 (4), 309–310. 10.1007/s12098-017-2455-5 28895042

[B38] Stuppia L. Antonucci I. Palka G. Gatta V. (2012). Use of the MLPA assay in the molecular diagnosis of gene copy number alterations in human genetic diseases. Int. J. Mol. Sci. 13 (3), 3245–3276. 10.3390/ijms13033245 22489151 PMC3317712

[B39] Suh M. R. Lee K. A. Kim E. Y. Jung J. Choi W. A. Kang S. W. (2017). Multiplex ligation-dependent probe amplification in X-linked recessive muscular dystrophy in korean subjects. Yonsei Med. J. 58 (3), 613–618. 10.3349/ymj.2017.58.3.613 28332368 PMC5368148

[B40] Takeshima Y. Yagi M. Okizuka Y. Awano H. Zhang Z. Yamauchi Y. (2010). Mutation spectrum of the dystrophin gene in 442 Duchenne/Becker muscular dystrophy cases from one Japanese referral center. J. Hum. Genet. 55 (6), 379–388. 10.1038/jhg.2010.49 20485447

[B41] Taylor P. J. Maroulis S. Mullan G. L. Pedersen R. L. Baumli A. Elakis G. (2007). Measurement of the clinical utility of a combined mutation detection protocol in carriers of Duchenne and Becker muscular dystrophy. J. Med. Genet. 44 (6), 368–372. 10.1136/jmg.2006.047464 17259292 PMC2740880

[B42] Vogel H. Zamecnik J. (2005). Diagnostic immunohistology of muscle diseases. J Neuropathol. Exp. Neurol. 64 (3), 181–193. 10.1093/jnen/64.3.181 15804049

[B43] Wang X. Wang Z. Yan M. Huang S. Chen T. J. Zhong N. (2008). Similarity of DMD gene deletion and duplication in the Chinese patients compared to global populations. Behav. Brain Funct. 4, 20. 10.1186/1744-9081-4-20 18445268 PMC2386868

[B44] Wang Y. Yang Y. Liu J. Chen X. C. Liu X. Wang C. Z. (2014). Whole dystrophin gene analysis by next-generation sequencing: a comprehensive genetic diagnosis of Duchenne and Becker muscular dystrophy. Mol. Genet. Genomics 289 (5), 1013–1021. 10.1007/s00438-014-0847-z 24770780

[B45] Wang L. Xu M. Li H. He R. Lin J. Zhang C. (2019). Genotypes and phenotypes of DMD small mutations in chinese patients with dystrophinopathies. Front. Genet. 10, 114. 10.3389/fgene.2019.00114 30833962 PMC6388391

[B46] Wein N. Alfano L. Flanigan K. M. (2015). Genetics and emerging treatments for Duchenne and Becker muscular dystrophy. Pediatr. Clin. North Am. 62 (3), 723–742. 10.1016/j.pcl.2015.03.008 26022172

[B47] Xiong H. Tan D. Wang S. Song S. Yang H. Gao K. (2015). Genotype/phenotype analysis in Chinese laminin-alpha2 deficient congenital muscular dystrophy patients. Clin. Genet. 87 (3), 233–243. 10.1111/cge.12366 24611677

[B48] Yiu E. M. Kornberg A. J. (2015). Duchenne muscular dystrophy. J. Paediatr. Child Health 51 (8), 759–764. 10.1111/jpc.12868 25752877

[B49] Zhang K. Yang X. Lin G. Han Y. Li J. (2019). Molecular genetic testing and diagnosis strategies for dystrophinopathies in the era of next generation sequencing. Clin. Chim. Acta 491, 66–73. 10.1016/j.cca.2019.01.014 30660698

[B50] Zimowski J. G. Pawelec M. Purzycka J. K. Szirkowiec W. Zaremba J. (2017). Deletions, not duplications or small mutations, are the predominante new mutations in the dystrophin gene. J. Hum. Genet. 62 (10), 885–888. 10.1038/jhg.2017.70 28680110

