# Supplementary material for: Molecular Genetics Analysis of 70 Chinese Families With Muscular Dystrophy Using Multiplex Ligation-Dependent Probe Amplification and Next-Generation Sequencing
Source: Front Pharmacol. 2019 Jul 25;10:814. doi: 10.3389/fphar.2019.00814 (PMC6669794; doi:10.3389/fphar.2019.00814)
Supplement: Supplementary file 1 [file Table_1.docx]

## Case information

| Patient ID | Age  (years) | Gender | Family history | Gene | Country | SNV or SV  (hg19 / GRCh37) | Disease name | Phenotype | Symptom |
| --- | --- | --- | --- | --- | --- | --- | --- | --- | --- |
| 1 | 0.2 | Male | No | *DMD* | China | ChrX:  g. (?_31140035)_(33357726_?)del  hemizygous  NM_004006.2,  NP_003997.1  del exon 1-79  p.0 | Duchenne’s muscular dystrophy or Becker muscular dystrophy | HP:0003236\|Elevated serum creatine phosphokinase  HP:0003458\|EMG: myopathic abnormalities | Muscular dystrophy; Duchenne’s; *DMD* MLPA |
| 2 | 7.9 | Male | No | *DMD* | China | ChrX:  g. (32867938_33038255)_(33038318_33229398)del  hemizygous  NM_004006.2,  NP_003997.1  del exon 2  p.( fs*?) | Duchenne’s muscular dystrophy | HP:0002355\|Difficulty walking  HP:0003391\|Gowers sign  HP:0003701\|Proximal muscle weakness  HP:0030235\|Extremely elevated creatine phosphokinase  HP:0003707\|Calf muscle pseudohypertrophy | Muscular dystrophy; Duchenne’s; *DMD* MLPA |
| 3 | 7.3 | Male | No | *DMD* | China | ChrX:  g. (32591964_32613873)_(32867938_33038255)del  hemizygous  NM_004006.2,  NP_003997.1  del exon 2-13  p.(del?) | Duchenne’s muscular dystrophy | HP:0002355\|Difficulty walking  HP:0003391\|Gowers sign  HP:0003551\|Difficulty climbing stairs  HP:0003701\|Proximal muscle weakness  HP:0030235\|Extremely elevated creatine phosphokinase  HP:0003707\|Calf muscle pseudohypertrophy | Muscular dystrophy; Duchenne’s; *DMD* MLPA |
| 4 | 1.8 | Male | Yes | *DMD* | China | ChrX:  g. (32430031_32456357)_(32867938_33038255)del  hemizygous  NM_004006.2,  NP_003997.1  del exon 3-29  p.(del?) | Duchenne’s muscular dystrophy | HP:0003391\|Gowers sign  HP:0030235\|Extremely elevated creatine phosphokinase | Muscular dystrophy; Duchenne’s; *DMD* MLPA |
| 5 | 2.4 | Female | No | *DMD* | China | ChrX:  g. (32613994_32632419)_(32717411_32827609)del  heterozygous  NM_004006.2,  NP_003997.1  del exon 8-12  p.(fs*) | Duchenne’s muscular dystrophy -like | HP:0003323\|Progressive muscle weakness  HP:0003236\|Elevated serum creatine phosphokinase | Muscular dystrophy; Duchenne’s; *DMD* MLPA |
| 6 | 8.0 | Male | No | *DMD* | China | ChrX:  g. (32482817_32486614)_(32717411_32827609)del  hemizygous  NM_004006.2,  NP_003997.1  del exon 8-23  p.(fs*) | Duchenne’s muscular dystrophy | HP:0002355\|Difficulty walking  HP:0001252\|Muscular hypotonia  HP:0002515\|Waddling gait  HP:0002527\|Falls  HP:0003551\|Difficulty climbing stairs  HP:0003391\|Gowers sign  HP:0030235\|Extremely elevated creatine phosphokinase | Muscular dystrophy; Duchenne’s; *DMD* MLPA |
| 7 | 2.7 | Male | No | *DMD* | China | ChrX:  g. (31986632_32235032)_(32663270_32715986)del  hemizygous  NM_004006.2,  NP_003997.1  del exon 10-44  p.(del) | Duchenne’s muscular dystrophy | HP:0030235\|Extremely elevated creatine phosphokinase  HP:0003458\|EMG: myopathic abnormalities  HP:0001252\|Muscular hypotonia  HP:0003707\|Calf muscle pseudohypertrophy | Muscular dystrophy; Duchenne’s; *DMD* MLPA |
| 8 | 2.9 | Male | No | *DMD* | China | ChrX:  g. (32591964_32613873)_(32613994_32632419)del  hemizygous  NM_004006.2,  NP_003997.1  del exon 13  p.(del) | Duchenne’s muscular dystrophy | HP:0003236\|Elevated serum creatine phosphokinase  HP:0003458\|EMG: myopathic abnormalities  HP:0003323\|Progressive muscle weakness | Muscular dystrophy; Duchenne’s; *DMD* MLPA |
| 9 | 1.7 | Male | Don’t know | *DMD* | China | ChrX:  g. (32383317_32398626)_(32472950_32481555)del  hemizygous  NM_004006.2,  NP_003997.1  del exon 26-34  p.(del) | Duchenne’s muscular dystrophy or Becker muscular dystrophy | HP:0003236\|Elevated serum creatine phosphokinase | Muscular dystrophy; Duchenne’s or Becker; *DMD* MLPA |
| 10 | 0.4 | Male | No | *DMD* | China | ChrX:  g. (31986632_32235032)_(32235181_32305645)del  hemizygous  NM_004006.2,  NP_003997.1  del exon 44  p.(fs*) | Duchenne’s muscular dystrophy or Becker muscular dystrophy | HP:0030235\|Extremely elevated creatine phosphokinase | Muscular dystrophy; Duchenne’s or Becker; *DMD* MLPA |
| 11 | 0.8 | Male | No | *DMD* | China | ChrX:  g. (31525571_31645789)_(32235181_32305645)del  hemizygous  NM_004006.2,  NP_003997.1  del exon 44-55  p.(fs*) | Duchenne’s muscular dystrophy or Becker muscular dystrophy | HP:0003236\|Elevated serum creatine phosphokinase | Muscular dystrophy; Duchenne’s or Becker; *DMD* MLPA |
| 12 | 0.6 | Male | No | *DMD* | China | ChrX:  g. (31950345_31986455)_(31986632_32235032)del  hemizygous  NM_004006.2,  NP_003997.1  del exon 45  p.(fs*) | Duchenne’s muscular dystrophy or Becker muscular dystrophy | HP:0030235\|Extremely elevated creatine phosphokinase | Muscular dystrophy; Duchenne’s or Becker; *DMD* MLPA |
| 13 | 7.3 | Male | No | *DMD* | China | ChrX:  g. (31950345_31986455)_(31986632_32235032)del  hemizygous  NM_004006.2,  NP_003997.1  del exon 45  p.(fs*) | Duchenne’s muscular dystrophy | HP:0002355\|Difficulty walking  HP:0002515 \|Waddling gait  HP:0002527\|Falls  HP:0003551\|Difficulty climbing stairs  HP:0003391\|Gowers sign  HP:0030235\|Extremely elevated creatine phosphokinase  HP:0003458\|EMG: myopathic abnormalities | Muscular dystrophy; Duchenne’s; *DMD* MLPA |
| 14 | 3.7 | Male | No | *DMD* | China | ChrX:  g. (31893491_31947712)_(31986632_32235032)del  hemizygous  NM_004006.2,  NP_003997.1  del exon 45-47  p.(del) | Becker muscular dystrophy | HP:0003236\|Elevated serum creatine phosphokinase  HP:0003707\|Calf muscle pseudohypertrophy | Muscular dystrophy; Becker; *DMD* MLPA |
| 15 | 1.0 | Male | No | *DMD* | China | ChrX:  g. (31893491_31947712)_(31986632_32235032)del  hemizygous  NM_004006.2,  NP_003997.1  del exon 45-47  p.(del) | Duchenne’s muscular dystrophy or Becker muscular dystrophy | HP:0003236\|Elevated serum creatine phosphokinase | Muscular dystrophy; Duchenne’s or Becker; *DMD* MLPA |
| 16 | 9.9 | Male | Don’t know | *DMD* | China | ChrX:  g. (31893491_31947712)_(31986632_32235032)del  hemizygous  NM_004006.2,  NP_003997.1  del exon 45-47  p.(del) | Becker muscular dystrophy | HP:0003551\|Difficulty climbing stairs  HP:0003707\|Calf muscle pseudohypertrophy  HP:0003236\|Elevated serum creatine phosphokinase | Muscular dystrophy; Becker; *DMD* MLPA |
| 17 | 5.3 | Male | No | *DMD* | China | ChrX:  g. (31854937_31893304)_(31986632_32235032)del  hemizygous  NM_004006.2,  NP_003997.1  del exon 45-48  p.(del) | Becker muscular dystrophy | HP:0003707\|Calf muscle pseudohypertrophy  HP:0003236\|Elevated serum creatine phosphokinase  HP:0003458\|EMG: myopathic abnormalities | Muscular dystrophy; Becker; *DMD* MLPA |
| 18 | 7.6 | Male | No | *DMD* | China | ChrX:  g. (31645980_31676106)_(31986632_32235032)del  hemizygous  NM_004006.2,  NP_003997.1  del exon 45-54  p.( fs*) | Duchenne’s muscular dystrophy | HP:0003323\|Progressive muscle weakness  HP:0002527\|Falls  HP:0030235\|Extremely elevated creatine phosphokinase  HP:0002515 \|Waddling gait | Muscular dystrophy; Duchenne’s; *DMD* MLPA |
| 19 | 1.8 | Male | No | *DMD* | China | ChrX:  g. (31645980_31676106)_(31986632_32235032)del  hemizygous  NM_004006.2,  NP_003997.1  del exon 45-54  p.( fs*) | Duchenne’s muscular dystrophy | HP:0003323\|Progressive muscle weakness  HP:0030235\|Extremely elevated creatine phosphokinase  HP:0003458\|EMG: myopathic abnormalities | Muscular dystrophy; Duchenne’s; *DMD* MLPA |
| 20 | 0.6 | Male | No | *DMD* | China | ChrX:  g. (31645980_31676106)_(31986632_32235032)del  hemizygous  NM_004006.2,  NP_003997.1  del exon 45-54  p.( fs*) | Duchenne’s muscular dystrophy or Becker muscular dystrophy | HP:0030235\|Extremely elevated creatine phosphokinase  HP:0003458\|EMG: myopathic abnormalities | Muscular dystrophy; Duchenne’s or Becker; *DMD* MLPA |
| 21 | 0.1 | Male |  | *DMD* | China | ChrX:  g. (31854937_31893304)_(31950345_31986455)del  hemizygous  NM_004006.2,  NP_003997.1  del exon 46-48  p.( fs*) | Duchenne’s muscular dystrophy or Becker muscular dystrophy | HP:0030235\|Extremely elevated creatine phosphokinase  HP:0003458\|EMG: myopathic abnormalities | Muscular dystrophy; Duchenne’s or Becker; *DMD* MLPA |
| 22 | 0.9 | Male | No | *DMD* | China | ChrX:  g. (31697704_31747747)_(31950345_31986455)del  hemizygous  NM_004006.2,  NP_003997.1  del exon 46-52  p.( fs*) | Duchenne’s muscular dystrophy or Becker muscular dystrophy | HP:0003707\|Calf muscle pseudohypertrophy  HP:0003236\|Elevated serum creatine phosphokinase  HP:0003458\|EMG: myopathic abnormalities | Muscular dystrophy; Duchenne’s or Becker; *DMD* MLPA |
| 23 | 6.2 | Male | No | *DMD* | China | ChrX:  g. (31525571_31645789)_(31950345_31986455)del  hemizygous  NM_004006.2,  NP_003997.1  del exon 46-55  p.( fs*) | Duchenne’s muscular dystrophy | HP:0002355\|Difficulty walking  HP:0003551\|Difficulty climbing stairs  HP:0003707\|Calf muscle pseudohypertrophy  HP:0003323\|Progressive muscle weakness  HP:0030235\|Extremely elevated creatine phosphokinase  HP:0003458\|EMG: myopathic abnormalities | Muscular dystrophy; Duchenne’s; *DMD* MLPA |
| 24 | 0.1 | Male | Yes | *DMD* | China | ChrX:  g. (31525571_31645789)_(31950345_31986455)del  hemizygous  NM_004006.2,  NP_003997.1  del exon 46-55  p.( fs*) | Duchenne’s muscular dystrophy or Becker muscular dystrophy | HP:0003236\|Elevated serum creatine phosphokinase  HP:0003458\|EMG: myopathic abnormalities | Muscular dystrophy; Duchenne’s or Becker; *DMD* MLPA |
| 25 | 7.0 | Male | No | *DMD* | China | ChrX:  g. (31792310_31838091)_(31893491_31947712)del  hemizygous  NM_004006.2,  NP_003997.1  del exon 48-50  p.( fs*) | Duchenne’s muscular dystrophy | HP:0002515\|Waddling gait  HP:0003551\|Difficulty climbing stairs  HP:0003391\|Gowers sign  HP:0030235\|Extremely elevated creatine phosphokinase | Muscular dystrophy; Duchenne’s; *DMD* MLPA |
| 26 | 1.7 | Male | No | *DMD* | China | ChrX:  g. (31792310_31838091)_(31854937_31893304)del  hemizygous  NM_004006.2,  NP_003997.1  del exon 49-50  p.( fs*) | Duchenne’s muscular dystrophy or Becker muscular dystrophy | HP:0030235\|Extremely elevated creatine phosphokinase  HP:0003458\|EMG: myopathic abnormalities | Muscular dystrophy; Duchenne’s or Becker; *DMD* MLPA |
| 27 | 9.0 | Male | No | *DMD* | China | ChrX:  g. (31792310_31838091)_(31854937_31893304)del  hemizygous  NM_004006.2,  NP_003997.1  del exon 49-50  p.( fs*) | Duchenne’s muscular dystrophy | HP:0003551\|Difficulty climbing stairs  HP:0003391\|Gowers sign  HP:0002527\|Falls  HP:0030235\|Extremely elevated creatine phosphokinase  HP:0002515\|Waddling gait | Muscular dystrophy; Duchenne’s; *DMD* MLPA |
| 28 | 0.7 | Male | No | *DMD* | China | ChrX:  g. (31697704_31747747)_(31854937_31893304)del  hemizygous  NM_004006.2,  NP_003997.1  del exon 49-52  p.( fs*) | Duchenne’s muscular dystrophy or Becker muscular dystrophy | HP:0003236\|Elevated serum creatine phosphokinase  HP:0003458\|EMG: myopathic abnormalities | Muscular dystrophy; Duchenne’s or Becker; *DMD* MLPA |
| 29 | 0.1 | Male | Yes | *DMD* | China | ChrX:  g. (31697704_31747747)_(31854937_31893304)del  hemizygous  NM_004006.2,  NP_003997.1  del exon 49-52  p.( fs*) | Duchenne’s muscular dystrophy or Becker muscular dystrophy | HP:0003236\|Elevated serum creatine phosphokinase | Muscular dystrophy; Duchenne’s or Becker; *DMD* MLPA |
| 30 | 8.0 | Female | No | *DMD* | China | ChrX:  g. (31792310_31838091)_(31838201_31854834)del  heterozygous  NM_004006.2,  NP_003997.1  del exon 50  p.( fs*) | Duchenne’s muscular dystrophy | HP:0003323\|Progressive muscle weakness  HP:0008981\|Calf muscle hypertrophy  HP:0003236\|Elevated serum creatine phosphokinase | Muscular dystrophy; Duchenne’s; *DMD* MLPA |
| 31 | 1.1 | Male | No | *DMD* | China | ChrX:  g. (31697704_31747747)_(31838201_31854834)del  hemizygous  NM_004006.2,  NP_003997.1  del exon 50-52  p.( fs*) | Duchenne’s muscular dystrophy | HP:0002355\|Difficulty walking  HP:0003236\|Elevated serum creatine phosphokinase | Muscular dystrophy; Duchenne’s; *DMD* MLPA |
| 32 | 3.0 | Male | No | *DMD* | China | ChrX:  g. (31697704_31747747)_(31838201_31854834)del  hemizygous  NM_004006.2,  NP_003997.1  del exon 50-52  p.( fs*) | Duchenne’s muscular dystrophy | HP:0003323\|Progressive muscle weakness  HP:0003391\|Gowers sign  HP:0030235\|Extremely elevated creatine phosphokinase | Muscular dystrophy; Duchenne’s; *DMD* MLPA |
| 33 | 1.2 | Male | No | *DMD* | China | ChrX:  g. (31747866_31792076)_(31792310_31838091)del  hemizygous  NM_004006.2,  NP_003997.1  del exon 51  p.( fs*) | Duchenne’s muscular dystrophy or Becker muscular dystrophy | HP:0030235\|Extremely elevated creatine phosphokinase  HP:0003458\|EMG: myopathic abnormalities | Muscular dystrophy; Duchenne’s or Becker; *DMD* MLPA |
| 34 | 0.7 | Male | No | *DMD* | China | ChrX:  g. (31747866_31792076)_(31792310_31838091)del  hemizygous  NM_004006.2,  NP_003997.1  del exon 51  p.( fs*) | Duchenne’s muscular dystrophy or Becker muscular dystrophy | HP:0030235\|Extremely elevated creatine phosphokinase  HP:0003458\|EMG: myopathic abnormalities | Muscular dystrophy; Duchenne’s or Becker; *DMD* MLPA |
| 35 | 9.0 | Male | No | *DMD* | China | ChrX:  g. (31747866_31792076)_(31792310_31838091)del  hemizygous  NM_004006.2,  NP_003997.1  del exon 51  p.( fs*) | Duchenne’s muscular dystrophy | HP:0003323\|Progressive muscle weakness  HP:0003551\|Difficulty climbing stairs  HP:0030235\|Extremely elevated creatine phosphokinase  HP:0003391\|Gowers sign  HP:0003458\|EMG: myopathic abnormalities | Muscular dystrophy; Duchenne’s; *DMD* MLPA |
| 36 | 7.0 | Male | No | *DMD* | China | ChrX:  g. (31747866_31792076)_(31792310_31838091)del  hemizygous  NM_004006.2,  NP_003997.1  del exon 51  p.( fs*) | Duchenne’s muscular dystrophy | HP:0030235\|Extremely elevated creatine phosphokinase  HP:0003707\|Calf muscle pseudohypertrophy  HP:0003391\|Gowers sign | Muscular dystrophy; Duchenne’s; *DMD* MLPA |
| 37 | 3.2 | Male | No | *DMD* | China | ChrX:  g. (31525571_31645789)_(31792310_31838091)del  hemizygous  NM_004006.2,  NP_003997.1  del exon 51-55  p.( fs*) | Duchenne’s muscular dystrophy | HP:0003458\|EMG: myopathic abnormalities  HP:0030235\|Extremely elevated creatine phosphokinase  HP:0003707\|Calf muscle pseudohypertrophy | Muscular dystrophy; Duchenne’s; *DMD* MLPA |
| 38 | 7.5 | Male | No | *DMD* | China | ChrX:  g. (31525571_31645789)_(31792310_31838091)del  hemizygous  NM_004006.2,  NP_003997.1  del exon 51-55  p.( fs*) | Duchenne’s muscular dystrophy | HP:0002355\|Difficulty walking  HP:0003551\|Difficulty climbing stairs  HP:0003391\|Gowers sign  HP:0003323\|Progressive muscle weakness  HP:0003707\|Calf muscle pseudohypertrophy  HP:0030235\|Extremely elevated creatine phosphokinase | Muscular dystrophy; Duchenne’s; *DMD* MLPA |
| 39 | 1.0 | Male | No | *DMD* | China | ChrX:  g. (31697704_31747747)_(31747866_31792076)del  hemizygous  NM_004006.2,  NP_003997.1  del exon 52  p.( fs*) | Duchenne’s muscular dystrophy or Becker muscular dystrophy | HP:0003458\|EMG: myopathic abnormalities  HP:0003236\|Elevated serum creatine phosphokinase | Muscular dystrophy; Duchenne’s or Becker; *DMD* MLPA |
| 40 | 0.6 | Male | No | *DMD* | China | ChrX:  g. (31676262_31697491)_(31697704_31747747)del  hemizygous  NM_004006.2,  NP_003997.1  del exon 53  p.( fs*) | Duchenne’s muscular dystrophy or Becker muscular dystrophy | HP:0003458\|EMG: myopathic abnormalities  HP:0003236\|Elevated serum creatine phosphokinase | Muscular dystrophy; Duchenne’s or Becker; *DMD* MLPA |
| 41 | 4.0 | Male | No | *DMD* | China | ChrX:  g. (31525571_31645789)_(31697704_31747747)del  hemizygous  NM_004006.2,  NP_003997.1  del exon 53-55  p.( fs*) | Duchenne’s muscular dystrophy | HP:0003236\|Elevated serum creatine phosphokinase  HP:0003458\|EMG: myopathic abnormalities  HP:0003323\|Progressive muscle weakness | Muscular dystrophy; Duchenne’s; *DMD* MLPA |
| 42 | 0.8 | Male | No | *DMD* | China | ChrX:  g. (31525571_31645789)_(31645980_31676106)del  hemizygous  NM_004006.2,  NP_003997.1  del exon 55  p.( fs*) | Duchenne’s muscular dystrophy or Becker muscular dystrophy | HP:0030235\|Extremely elevated creatine phosphokinase  HP:0003458\|EMG: myopathic abnormalities | Muscular dystrophy; Duchenne’s or Becker; *DMD* MLPA |
| 43 | 1.8 | Male | No | *DMD* | China | ChrX:  g. (31144791_31152218)_(31645980_31676106)del  hemizygous  NM_004006.2,  NP_003997.1  del exon 55-77  p.( fs*) | Duchenne’s muscular dystrophy | HP:0003391\|Gowers sign  HP:0030235\|Extremely elevated creatine phosphokinase  HP:0003458\|EMG: myopathic abnormalities | Muscular dystrophy; Duchenne’s; *DMD* MLPA |
| 44 | 2.0 | Male | No | *DMD* | China | ChrX:  g. _31140035)_(31525571_31645789)del  hemizygous  NM_004006.2,  NP_003997.1  del exon 56-79  p.? | Duchenne’s muscular dystrophy | HP:0003236\|Elevated serum creatine phosphokinase  HP:0002194\|Delayed gross motor development | Muscular dystrophy; Duchenne’s; *DMD* MLPA |
| 45 | 0.7 | Male | No | *DMD* | China | ChrX:  g. (31341776_31366672)_(31366752_31462597)del  hemizygous  NM_004006.2,  NP_003997.1  del exon 61  p.( fs*) | Duchenne’s muscular dystrophy or Becker muscular dystrophy | HP:0003236\|Elevated serum creatine phosphokinase  HP:0003458\|EMG: myopathic abnormalities | Muscular dystrophy; Duchenne’s or Becker; *DMD* MLPA |
| 46 | 6.7 | Male | No | *DMD* | China | ChrX:  g. (31279134_31341714)_(31341776_31366672)del  hemizygous  NM_004006.2,  NP_003997.1  del exon 62  p.( fs*) | Duchenne’s muscular dystrophy | HP:0003323\|Progressive muscle weakness  HP:0002515\|Waddling gait  HP:0003707\|Calf muscle pseudohypertrophy  HP:0003391\|Gowers sign  HP:0030235\|Extremely elevated creatine phosphokinase  HP:0003458\|EMG: myopathic abnormalities | Muscular dystrophy; Duchenne’s; *DMD* MLPA |
| 47 | 0.1 | Male | No | *DMD* | China | ChrX:  g. (31191722_31196048) _(31198599_31200854)del  hemizygous  NM_004006.2,  NP_003997.1  del exon 69-71  p.( fs*) | Duchenne’s muscular dystrophy or Becker muscular dystrophy | HP:0030235\|Extremely elevated creatine phosphokinase  HP:0003458\|EMG: myopathic abnormalities | Muscular dystrophy; Duchenne’s or Becker; *DMD* MLPA |
| 48 | 7.4 | Male | No | *DMD* | China | ChrX:  g. (32717411_32827609)_(32867938_33038255)dup  hemizygous  NM_004006.2,  NP_003997.1  dup exon 3-7  p.( fs*) | Duchenne’s muscular dystrophy | HP:0001324\|Muscle weakness  HP:0001270\|Motor delay  HP:0030235\|Extremely elevated creatine phosphokinase | Muscular dystrophy; Duchenne’s; *DMD* MLPA |
| 49 | 5.6 | Male | No | *DMD* | China | ChrX:  g. (32717411_32827609)_(32841505_32862899)dup  hemizygous  NM_004006.2,  NP_003997.1  dup exon 5-7  p.( fs*) | Duchenne’s muscular dystrophy | HP:0030235\|Extremely elevated creatine phosphokinase  HP:0003458\|EMG: myopathic abnormalities | Muscular dystrophy; Duchenne’s; *DMD* MLPA |
| 50 | 2.7 | Male | No | *DMD* | China | ChrX:  g. (32490427_32503035)_(32519960_32536124)dup  hemizygous  NM_004006.2,  NP_003997.1  dup exon 19-21  p.( fs*) | Duchenne’s muscular dystrophy | HP:0003323\|Progressive muscle weakness  HP:0003551\|Difficulty climbing stairs  HP:0003391\|Gowers sign  HP:0003707\|Calf muscle pseudohypertrophy  HP:0030235\|Extremely elevated creatine phosphokinase | Muscular dystrophy; Duchenne’s; *DMD* MLPA |
| 51 | 7.0 | Male | No | *DMD* | China | ChrX:  g. (31645980_31676106)_(31986632_32235032)dup  hemizygous  NM_004006.2,  NP_003997.1  dup exon 45-54  p.( fs*) | Duchenne’s muscular dystrophy | HP:0002355\|Difficulty walking  HP:0003391\|Gowers sign  HP:0003707\|Calf muscle pseudohypertrophy  HP:0003236\|Elevated serum creatine phosphokinase  HP:0003458\|EMG: myopathic abnormalities | Muscular dystrophy; Duchenne’s; *DMD* sequencing |
| 52 | 2.1 | Male | No | *DMD* | China | ChrX:  g. 32509580C>T,  hemizygous  NM_004006.2,  NP_003997.1  c.2436C>T,  p.(Trp812*) | Duchenne’s muscular dystrophy | HP:0002355\|Difficulty walking; HP:0003707\|Calf muscle pseudohypertrophy; HP:0003236\|Elevated serum creatine phosphokinase; HP:0003458\|EMG: myopathic abnormalities | Muscular dystrophy; Duchenne’s; *DMD* sequencing |
| 53 | 6.4 | Male | No | *DMD* | China | ChrX:  g. 31838137_31838138insG  hemizygous  NM_004006.2,  NP_003997.1  c.7263_7264insG,  p.(Ala2422Glyfs*5) | Duchenne’s muscular dystrophy | HP:0008981\|Calf muscle hypertrophy; HP:0003323\|Progressive muscle weakness; HP:0003236\|Elevated serum creatine phosphokinase | Muscular dystrophy; Duchenne’s; *DMD* sequencing |
| 54 | 4.0 | Male | No | *DMD* | China | ChrX:  g. 32662349A>T,  hemizygous  NM_004006.2,  NP_003997.1  c.1231A>T,  p.(Lys411*) | Duchenne’s muscular dystrophy | HP:0003323\|Progressive muscle weakness; HP:0003707\|Calf muscle pseudohypertrophy | Muscular dystrophy; Duchenne’s; *DMD* sequencing |
| 55 | 0.9 | Male | Yes | *DMD* | China | ChrX:  g. 32827679C>T,  hemizygous  NM_004006.2,  NP_003997.1  c.580C>T,  p.(Gln194*) | Duchenne’s muscular dystrophy or Becker muscular dystrophy | HP:0003236\|Elevated serum creatine phosphokinase; HP:0003458\|EMG: myopathic abnormalities; HP:0003707\|Calf muscle pseudohypertrophy | Muscular dystrophy; Duchenne’s or Becker; *DMD* sequencing |
| 56 | 3.8 | Male | No | *DMD* | China | ChrX:  g. 32591646C>T,  hemizygous  NM_004006.2,  NP_003997.1  c.1812+1C>T | Becker muscular dystrophy | HP:0003236\|Elevated serum creatine phosphokinase; HP:0003707\|Calf muscle pseudohypertrophy | Muscular dystrophy; Becker; *DMD* sequencing |
| 57 | 3.1 | Male | No | *DMD* | China | ChrX:  g. 32381063G>T,  hemizygous  NM_004006.2,  NP_003997.1  c.5167G>T  p. (Gln1723*) | Duchenne’s muscular dystrophy | HP:0003236\|Elevated serum creatine phosphokinase; HP:0003707\|Calf muscle pseudohypertrophy; HP:0003458\|EMG: myopathic abnormalities | Muscular dystrophy; Duchenne’s; *DMD* sequencing |
| 58 | 2.3 | Male | No | *DMD* | China | ChrX:  g. 31196822delC  hemizygous  NM_004006.2,  NP_003997.1  10187delC  p.(Pro3396Glnfs*6) | Duchenne’s muscular dystrophy | HP:0030235\|Extremely elevated creatine phosphokinase; HP:0003458\|EMG: myopathic abnormalities; HP:0002194\|Delayed gross motor development | Muscular dystrophy; Duchenne’s; *DMD* sequencing |
| 59 | 1.2 | Male | No | *DMD* | China | ChrX:  g. 31747747C>G,  hemizygous  NM_004006.2,  NP_003997.1  c.7660+1C>G | Duchenne’s muscular dystrophy or Becker muscular dystrophy | HP:0003236\|Elevated serum creatine phosphokinase | Muscular dystrophy; Duchenne’s or Becker; *DMD* sequencing |
| 60 | 1.0 | Male | No | *DMD* | China | ChrX:  g. 31697572 C>T,  hemizygous  NM_004006.2,  NP_003997.1  c.7792C>T  p.(Gln2598*) | Duchenne’s muscular dystrophy or Becker muscular dystrophy | HP:0030235\|Extremely elevated creatine phosphokinase | Muscular dystrophy; Duchenne’s or Becker; *DMD* sequencing |
| 61 | 7.0 | Male | No | *DMD* | China | ChrX:  g. 32509445delC,  hemizygous  NM_004006.2,  NP_003997.1  c.2571delC  p.(Pro857Profs*14) | Duchenne’s muscular dystrophy | HP:0002355\|Difficulty walking;  HP:0003391\|Gowers sign;  HP:0003707\|Calf muscle pseudohypertrophy;  HP:0030235\|Extremely elevated creatine phosphokinase | Muscular dystrophy; Duchenne’s; *DMD* sequencing |
| 62 | 1.0 | Male | No | *DMD* | China | ChrX:  g. 32305653C>T,  hemizygous  NM_004006.2,  NP_003997.1  c.6283C>T  p.(Arg2095*) | Duchenne’s muscular dystrophy or Becker muscular dystrophy | HP:0003236\|Elevated serum creatine phosphokinase;  HP:0003707\|Calf muscle pseudohypertrophy | Muscular dystrophy; Duchenne’s or Becker; *DMD* sequencing |
| 63 | 0.6 | Female | No | *LAMA2* | China | chr6:  g. 129573393-129573394delAG,  heterozygous  NM_000426.3,  NP_000417.2  c.2049_2050delAG  p.(Arg683Serfs*21) and  chr6:  g. (129381042_129419317)_(129419561_129465045)del  heterozygous  del exon 4  p.(del?) | Muscular dystrophy, congenital, merosin deficient or partially deficient | HP:0001324\|Muscle weakness;  HP:0001252\|Muscular hypotonia;  HP:0001270\|Motor delay;  HP:0002015\|Dysphagia;  HP:0030234 Highly elevated creatine phosphokinase;  HP:0001612\|Weak cry;  HP:0003458\|EMG: myopathic abnormalities | Muscular dystrophy; congenital; *LAMA2* sequencing and MLPA |
| 64 | 0.4 | Male | No | *LAMA2* | China | chr6:  g. 129513888C>T,  heterozygous  NM_000426.3,  NP_000417.2  c.1672C>T  p.(Gln558*) and  chr6:  g. (129381042_129419317)_(129419561_129465045)del  heterozygous  del exon 4  p.(del?) | Muscular dystrophy, congenital, merosin deficient or partially deficient | HP:0001324\|Muscle weakness;  HP:0001252\|Muscular hypotonia;  HP:0001270\|Motor delay;  HP:0002015\|Dysphagia;  HP:0030234\|Highly elevated creatine phosphokinase;  HP:0001612\|Weak cry;  HP:0003458\|EMG: myopathic abnormalities | Muscular dystrophy; congenital; *LAMA2* sequencing and MLPA |
| 65 | 5.6 | Male | No | *DMD* | China | No mutation was found | Becker muscular dystrophy | HP:0030235\|Extremely elevated creatine phosphokinase  HP:0003707\|Calf muscle pseudohypertrophy  HP:0003458\|EMG: myopathic abnormalities | Muscular dystrophy; Becker |
| 66 | 1.7 | Male | No | *DMD* | China | No mutation was found | Duchenne’s muscular dystrophy or Becker muscular dystrophy | HP:0003236\|Elevated serum creatine phosphokinase | Muscular dystrophy; Duchenne’s or Becker |
| 67 | 3.0 | Male | No | *DMD* | China | No mutation was found | Duchenne’s muscular dystrophy or Becker muscular dystrophy | HP:0003236\|Elevated serum creatine phosphokinase | Muscular dystrophy; Duchenne’s or Becker |
| 68 | 5.4 | Male | No | *DMD* | China | No mutation was found | Duchenne’s muscular dystrophy | HP:0003391\|Gowers sign  HP:0030235\|Extremely elevated creatine phosphokinase | Muscular dystrophy; Duchenne’s |
| 69 | 1.7 | Male | No | *DMD* | China | No mutation was found | Duchenne’s muscular dystrophy | HP:0003236\|Elevated serum creatine phosphokinase  HP:0003707\|Calf muscle pseudohypertrophy | Muscular dystrophy; Duchenne’s |
| 70 |  | Male | No | *DMD* | China | No mutation was found | Becker muscular dystrophy | HP:0003236\|Elevated serum creatine phosphokinase | Muscular dystrophy; Becker |

Del=deletion; Dup=duplication.
